# Subtotal Gastrectomy as “Last Resort” Consideration in the Management of Refractory Rumination Syndrome

**DOI:** 10.14740/gr594w

**Published:** 2014-07-31

**Authors:** Chad J. Cooper, Salman Otoukesh, Mona Mojtahedzadeh, Juan M. Galvis, Richard W. McCallum

**Affiliations:** a Department of Internal Medicine, Texas Tech University Health Sciences Center, El Paso, Texas, USA; b Department of Psychiatry, Texas Tech University Health Sciences Center, El Paso, Texas, USA

**Keywords:** Rumination syndrome, Subtotal gastrectomy, Vomiting, Diarrhea

## Abstract

Rumination syndrome is a behavioral disorder resulting in effortless regurgitation of undigested food within minutes of meal intake that is subsequently either re-swallowed or ejected. It is commonly misdiagnosed, patients often undergo extensive testing and multiple therapies, many of which are directed at suspected gastroparesis. A 25-year-old Caucasian female initially presented to our care 1 year ago with a 4-year history of nausea and vomiting occurring in the immediate postprandial period, specifically within 15 minutes from oral intake. She had an extensive history of multiple diagnostic, therapeutic and surgical procedures over the previous 4 years which included cholecystectomy, botulin toxin injection into the pyloric sphincter, pyloroplasty, placement of a gastric stimulator and jejunal feeding tube with no sustained results. On a previous admission we determined the functional status of the stomach by obtaining full thickness gastric biopsies during a diagnostic laparoscopy. This revealed an adequate population number of cells of Cajal and myenteric neurons as well as normal stomach muscle. After 1 year of attempting “breathing relaxation techniques”, while being nutritionally maintained by nocturnal jejunostomy feedings, the patient presented again to our care with refractory nausea and vomiting and unable to work or function. Her weight was 90 lbs. She underwent a subtotal gastrectomy (80%) with Roux-en-Y reconstruction and continuation of jejunostomy feeding. The refractory nausea and vomiting significantly improved over the 4 weeks after discharge and breathing exercises were continued. On subsequent follow-up visits over a 6-month course, the refractory nausea and vomiting had resolved by more than 85% with and improvement in her BMI and quality of life.The recommended treatment of rumination syndrome is focused on breathing exercises and relaxation techniques to “distract” while eating. We believe our case is the first reported where a subtotal gastrectomy has been used to help overcome refractory rumination along with the usual therapy. This surgery is a “last resort” consideration to improve quality of life, returning the patient to employment and functional social status.

## Introduction

Rumination syndrome is a behavioral disorder that consists of an effortless regurgitation of undigested food that is subsequently re-swallowed or ejected within minutes of meal ingestion. It is a chronic condition that typically occurs on a daily basis after every meal, occurring with both liquids and solids. The repetitive regurgitation of gastric contents starts typically within minutes and can persist for up to 1 - 2 h postprandial. The act of regurgitation in this condition is not a conscious decision but rather a reflex response. Rumination syndrome was first described in mentally handicapped children, but has increasingly been recognized among adolescents and adults of normal mental capacity.

The etiology and physiological mechanisms of this syndrome are not well understood. Patients with rumination syndrome seem to have a psychological component to their condition. Stressful life events may also play a role, and in some patients they have been identified around the period of symptom onset. These patients suffer a significant amount of functional disability related to extreme weight loss, absence from work or school, frequent hospitalizations and emergency department visits. Rumination syndrome is a complex disorder with symptoms that can mimic other gastroesophageal disorders. It is frequently confused with bulimia nervosa, gastroesophageal reflux disease and upper gastrointestinal motility disorders such as gastroparesis and chronic intestinal pseudo-obstruction [1]. The act of rumination is a consequence of a learned, voluntary relaxation of the lower esophageal sphincter or diaphragmatic crura. This allows the increased intragastric hydrostatic pressure in the postprandial period and tonic contractions of the proximal stomach to overcome the resistance to regurgitation usually provided by the antireflux mechanisms at the esophageal junction, leading to the retrograde movement of the gastric contents [2].

The diagnosis of rumination syndrome is based on the Rome III criteria (Table 1). Rumination syndrome is a clinical diagnosis made through a precise clinical history to distinguish it from other vomiting disorders. It involves the exclusion of other possible causes of regurgitation and/or postprandial vomiting. Unfortunately, many physicians lack the awareness of this condition and these patients undergo many diagnostic tests and invasive procedures that do not contribute to their management. In the case of diagnostic uncertainty, a manometric evaluation may confirm the diagnosis of rumination syndrome by revealing tall R-waves in gastric manometry tracings [3]. Studies to rule out other conditions include upper endoscopy, gastric emptying studies (GESs) and a 24-h esophageal pH monitoring. We present a case describing the chronic course of a patient with rumination syndrome that underwent multiple diagnostic tests and invasive procedures before finally being properly diagnosed and presenting to our care.

**Table 1:** Rome III Diagnostic Criteria for Rumination Syndrome: Adults

A. Obligatory criteria must include both of the following:
1. Persistent or recurrent regurgitation of recently ingested food with either mastication and swallowing or spitting out the regurgitated food.
2. Regurgitation is not preceded by retching.
B. Supportive criteria:
1. Regurgitation is not preceded by nausea.
2. Cessation when the regurgitated material becomes acidic.
3. Regurgitated material is recognizable food with a pleasant taste.
Criteria fulfilled for the last 3 months with symptom onset at least 6 months before diagnosis.

## Case Report

A 25-year-old Caucasian female presented to our care with a chronic history of regurgitation of previously ingested food. For the past 4 years, she regurgitated immediately in the postprandial period, occurring within 5 - 25 min after per oral (PO) intake and lasting up to 2 h. These acts of regurgitation were frequently observed by friends and family. When she presented to our care, she was very malnourished and had been maintained on nocturnal jejunal tube feedings. She also complained of multiple bouts of watery diarrhea, amounting to as much as ten episodes per day. Due to the repercussions of her illness, she avoided social gatherings and was not able to maintain her job due to these frequent episodes of regurgitation and subsequent vomiting or swallowing of the food.

Her past medical problems included asthma and anxiety. Since the beginning of her symptoms 4 years ago she was misdiagnosed multiple times with other gastrointestinal disorders. Therefore, she unnecessarily underwent many diagnostic studies and invasive surgical procedures. This downward spiral started when she was being evaluated for right upper quadrant abdominal pain associated with persistent episodes of vomiting. She subsequently had an abdominal ultrasound revealing biliary sludge and HIDA scan with an ejection fraction of < 20%. A laparoscopic cholecystectomy was performed in March 2010 based on the assumption that her condition was of biliary disease etiology. Her symptoms persisted despite this procedure, and idiopathic gastroparesis was the next assumption on their differentials of postprandial nausea and vomiting. The diagnosis of idiopathic gastroparesis was aided by a delayed GES. A unique finding from this evaluation was that some of the isotope was immediately regurgitated after being ingested. A normal upper endoscopy was also noted at this time. After failing medical therapy for gastroparesis, a gastric stimulator was placed in October of 2010 but with only < 10% improvement of symptoms. As a result, the patient underwent a pyloroplasty in January of 2011. Her symptoms improved for 2 - 3 weeks but then recurred and became associated with watery diarrhea. Her nutritional status deteriorated to the point that she eventually had a jejunostomy tube being placed in August of 2011.

After being referred to several gastroenterologists, she was eventually diagnosed to have rumination syndrome. To aid in the diagnosis, an antroduodenal manometry (ADM) was performed that revealed the characteristic R-waves exhibited in patients with rumination syndrome. The treatment of adult rumination syndrome consists of reassurance, behavior therapy, psychotherapy and relaxation therapies. All of these therapies were attempted and failed to provide a sustained relief of symptoms.

Physical examination findings revealed a cachectic female with a body mass index (BMI) of 16. Vital signs that were only significant for hypotension (BP: 80/55 mm Hg). She was in no acute distress with a normal mood, affect, attention span and concentration. Abdomen was soft, not distended, and not tender to palpation. A J-tube was located in the left upper quadrant and a gastric pacing device in the right upper quadrant, with no signs of infection. The remainder of the physical examination was unremarkable. There were no significant laboratory findings on admission.

The working diagnosis upon admission was rumination syndrome with conditioned vomiting. Our primary goal was to decrease the frequency of vomiting and diarrhea, which was eventually controlled with medical therapies of loperamide, dicycloamine, nortriptyline and scopolamine for nausea and pain control. Once the nausea, vomiting and diarrhea were better controlled, we gradually increased the rate of J-tube feeding to enhance her nutritional status. The general surgery service removed the gastric stimulator and replaced the feeding jejunostomy tube with a Mic-Key button. An upper endoscopy with subsequently done to obtain full thickness gastric biopsies for study of the gastric smooth muscle.

The surgical pathology results from the gastric biopsies revealed no evidence of inflammation, necrosis, intestinal metaplasia, dysplasia or malignancy. The nerve bundles and ganglion cells (myenteric plexus) were positive for S100 immunostain and were normal in number. An adequate population of cells of Cajal within the muscularis propria was indicated by positive C-kit staining. Ultimately these findings confirmed that the stomach was functionally normal at the molecular level. The patient was subsequently discharged with outpatient follow-up with a psychologist to put forth a dedicated effort in relaxation and other behavioral techniques.

After 1 year of dedicated attempts of “breathing relaxation techniques”, while being nutritionally maintained by nocturnal jejunostomy feedings, the patient presented again to our care with refractory nausea and vomiting. Her initial vital signs on admission were within normal limits. No significant physical examination findings were noted except she still weighed 90 lbs and had a BMI of 16. No significant abnormalities were noted on the initial laboratory work-up. An upper endoscopy was performed to evaluate the persistent vomiting and hematemesis. But no significant findings were noted except gastric mucosal atrophy. A GES was delayed with > 85% isotope retention. It was apparent that the stomach was anatomically normal but was not functionally normal.

Surgical intervention was considered as a last resort measure to improve the quality of life of the patient. An extensive psychiatrist evaluation was performed to rule out any psychiatric or eating disorders. The psychiatrist did not establish a diagnosis of anorexia or bulimia nervosa.

She underwent a subtotal gastrectomy (80%) with Roux-en-Y reconstruction on the fourth hospital day and the jejunostomy feeding was continued. Pathologic evaluation of the resected stomach revealed chronic gastritis, and the C-kit immunostain showed an average of 11 interstitial cells of Cajal (ICC) in the body of the stomach and 10 ICC in the antrum of the stomach. The jejunostomy tube feeding rate was gradually increased over the hospital course to a goal of 80 cc per hour and she was also tolerating a liquid diet. On subsequent follow-up visits over a 6-month course, the refractory nausea and vomiting had resolved by more than 85% with and improvement in her BMI and quality of life.

## Discussion

Approximately 17% of female patients diagnosed with rumination syndrome have been reported to have a history of bulimia [4]. One could speculate that it may be a learned behavior in which patients are able to purge themselves without digitally inducing frank vomiting. Alternatively, rumination might be thought of as a variant of bulimia nervosa or an atypical eating disorder [4].

It is hypothesized that patients with rumination syndrome use neural pathways to induce voluntary lower esophageal sphincter relaxation by abdominal wall contractions [5]. ADM shows brief simultaneous pressure increases (R-waves) associated with regurgitation, representing an involuntary act to increase the intra-abdominal pressure to induce regurgitation [6]. The timing of rumination can serve as a differentiating point from gastroparesis, in which vomiting is delayed for greater than 1 h after eating. Several diagnostic procedures that patient's with rumination syndrome commonly undergo include esophageal motility, upper gastroesophageal manometry, and gastric myoelectrical activity and GES. The results of these diagnostic procedures as established by a research study demonstrated that patents with rumination syndrome had a normal esophageal motility study, upper gastrointestinal motility with normal fasting and fed motility patterns, a gastric myoelectrical activity that had normal 2 - 4 cycles/min slow waves with no dysrhythmia and gastric emptying normal for a solid meal [7].

Currently, treatment of rumination syndrome is unsatisfactory in part because the pathophysiological mechanism is unclear [8]. Medications for rumination disease include acid blocking agents, prokinetic medications, antiemetics, anticholinergics, anxiolytics and antidepressants. These medications are for symptomatic relief and are not aimed at the underlying issues. Nissen fundoplication has been tried but has been reported to lead to complications like retching, bloating and gastroparesis [9].

Reassurance and behavioral therapy is currently the mainstay of treatment, with a reported success of 80%. Reassurance and education of the patient and the family are the first steps in the management. This involves providing a detailed explanation of the condition and handling expectations of the patient and family. This involves habit reversal by using several strategies such as positive encouragement not to vomit, biofeedback relaxation and diaphragmatic breathing.

Diaphragmatic breathing is the main technique taught to patients to reverse the habit of abdominal wall contraction and possibly alleviate symptoms of regurgitation associated with rumination syndrome [10]. The diaphragmatic breathing works by physically preventing the contraction of the abdominal wall that is required to regurgitate the gastric contents. The targeted behavior is therefore eliminated by the consistent use of the competing behavior. If this mechanism is performed on a consistent basis, the competing behavior will become dominant and therefore reduce or even eliminate the occurrence of regurgitation.

Recommended treatment of rumination syndrome is focused on breathing exercises and relaxation techniques to “distract” while eating. We believe our case is the first reported where a subtotal gastrectomy has been used to help overcome refractory rumination along with the usual therapy. This surgery is a “last resort” consideration to improve quality of life, returning the patient to employment and functional social status.
