# Corrigendum: Cooper et al, Subtotal Gastrectomy as “Last Resort” Consideration in the Management of Refractory Rumination Syndrome

**DOI:** 10.14740/gr624e

**Published:** 2014-12-27

**Authors:** Chad J. Cooper, Salman Otoukesh, Mona Mojtahedzadeh, Juan M. Galvis, Richard W. McCallum

**Affiliations:** a Department of Internal Medicine, Texas Tech University Health Sciences Center, El Paso, Texas, USA; b Department of Psychiatry, Texas Tech University Health Sciences Center, El Paso, Texas, USA

Corrections to article “Subtotal Gastrectomy as “Last Resort” Consideration in the Management of Refractory Rumination Syndrome”, by Chad J. Cooper et al, published in Vol. 7, No. 3-4, 2014, p98-101, doi: http://dx.doi.org/10.14740/gr594w.

The authors would like to make the following corrections.

In Abstract section, the sentence “On subsequent follow-up visits over a 6-month course, the refractory nausea and vomiting had resolved by more than 85% with and improvement in her BMI and quality of life.” should read “On subsequent follow-up visits over a 4-month course, the refractory nausea and vomiting had resolved by more than 70% with and improvement in her nutritional status and quality of life.”

In the last paragraph of Case Report section, the sentence “On subsequent follow-up visits over a 6-month course, the refractory nausea and vomiting had resolved by more than 85% with and improvement in her BMI and quality of life.” should read “On subsequent follow-up visits over a 4-month course, the refractory nausea and vomiting had resolved by more than 70% with and improvement in her nutritional status and quality of life.”

